# Tubulocystic renal cell carcinoma: A case report of rare tumor

**DOI:** 10.1016/j.radcr.2024.07.094

**Published:** 2024-08-13

**Authors:** Abdullah M. Al-Jubouri, Ibrahim A. Khalil, Abdelkareem Alhyari, Majd Alkabbani, Alaeddin Badawi, Rajen Goyal, Khalid Al Jalham

**Affiliations:** a College of Medicine, QU Health, Qatar University, Doha, Qatar; b Department of Urology, Uro-oncology section, Hamad Medical Corporation, Doha, Qatar; c Department of Laboratory Medicine and Pathology, Hamad Medical Corporation, Doha, Qatar; d Department of Urology, Hamad medical corporation, Doha, Qatar

**Keywords:** Tubulocystic, Renal cell carcinoma, Nephrectomy, Robotic

## Abstract

Tubulocystic renal cell carcinoma (RCC) is a rare renal cancer first recognized by the WHO in 2016 as independent disease category, characterized by its typically indolent features and low rates of metastasis. We present a 35-year-old male with tubulocystic RCC diagnosed incidentally on evaluation of flank pain. Magnetic resonance imaging showed Bosniak class 4 renal cyst, although initial computed topography showed a hypodense nonenhancing lesion classified as Bosniak 1 cyst. Patient underwent robotic assisted partial nephrectomy, histopathology confirmed as tubulocystic RCC. This case highlights the importance of considering tubulocystic RCC in the differential diagnoses of renal cysts and other solid renal masses to ensure timely and effective treatment plan.

## Introduction

Tubulocystic Renal Cell Carcinoma (RCC) is a rare entity of renal cancers that was first acknowledged by the World Health Organization (WHO) as a new variant of RCC in 2016 [1]. This subtype of renal cancers have proven that it can pose difficulty in diagnosis, especially when the total prevalence of this cancer worldwide is believed to be around 100 cases [2]. It is thought that this entity of tumors are usually indolent, showing low levels of clinical aggression, with metastasis rates being around 6% [3]. The latter explains the fact that most people achieve almost a normal quality of life after undergoing surgery for this tumor [2]. This rare entity has shown great levels of ambiguity with regards to clinical presentation, given the fact that most patients are actually asymptomatic and many of them are found incidentally via radiological scans for other reasons, such as renal cysts [4]. However, according to one study, this tumor entity is 7 times more likely to affect males compared to females, and the tumor usually affects the left kidney more frequently [5]. It is also crucial to mention that there has been no specific, well-recognized risk factors contributing to the occurrence of this tumor [5]. In this study, we present a case of a male patient who was diagnosed with tubulocystic RCC with challenging radiological evaluation.

## Case presentation

**Fig. 1:**
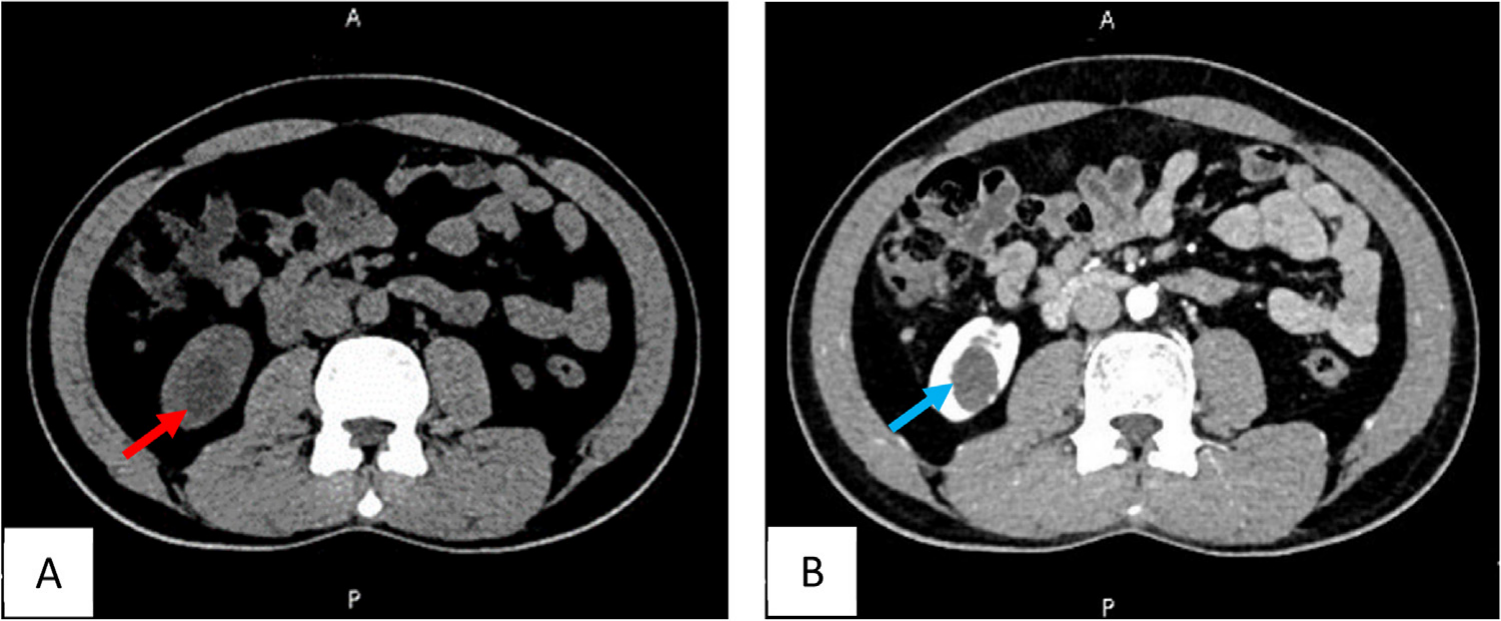
CT scan of the right renal lesion. (A) non contrast CT showing homogenous and hypodense lesion (red arrow), (B) no enhancement seen after IV contrast (blue arrow).

**Fig. 2:**
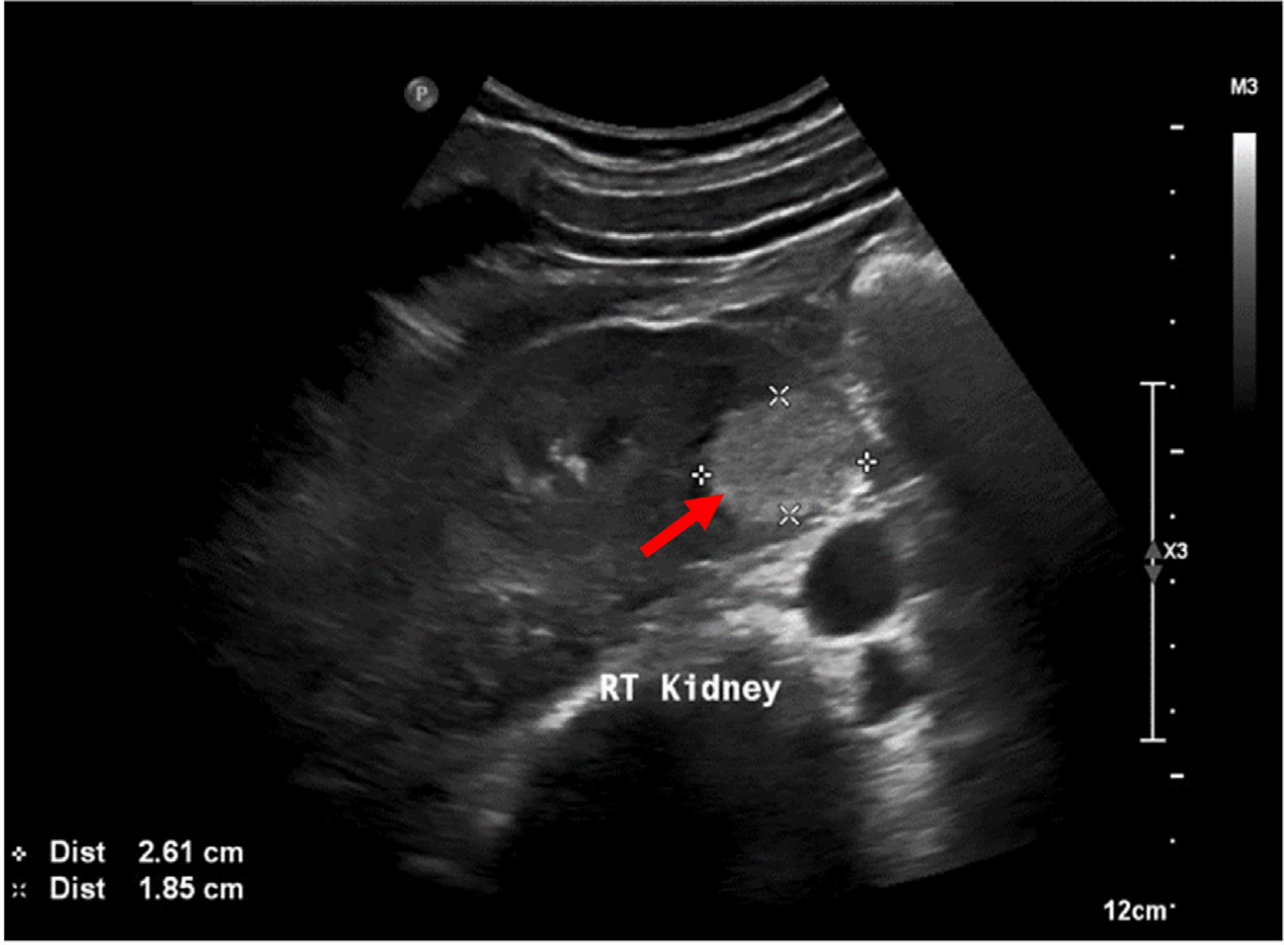
US showing hyperdense lesion in right kidney (red arrow).

**Fig. 3:**
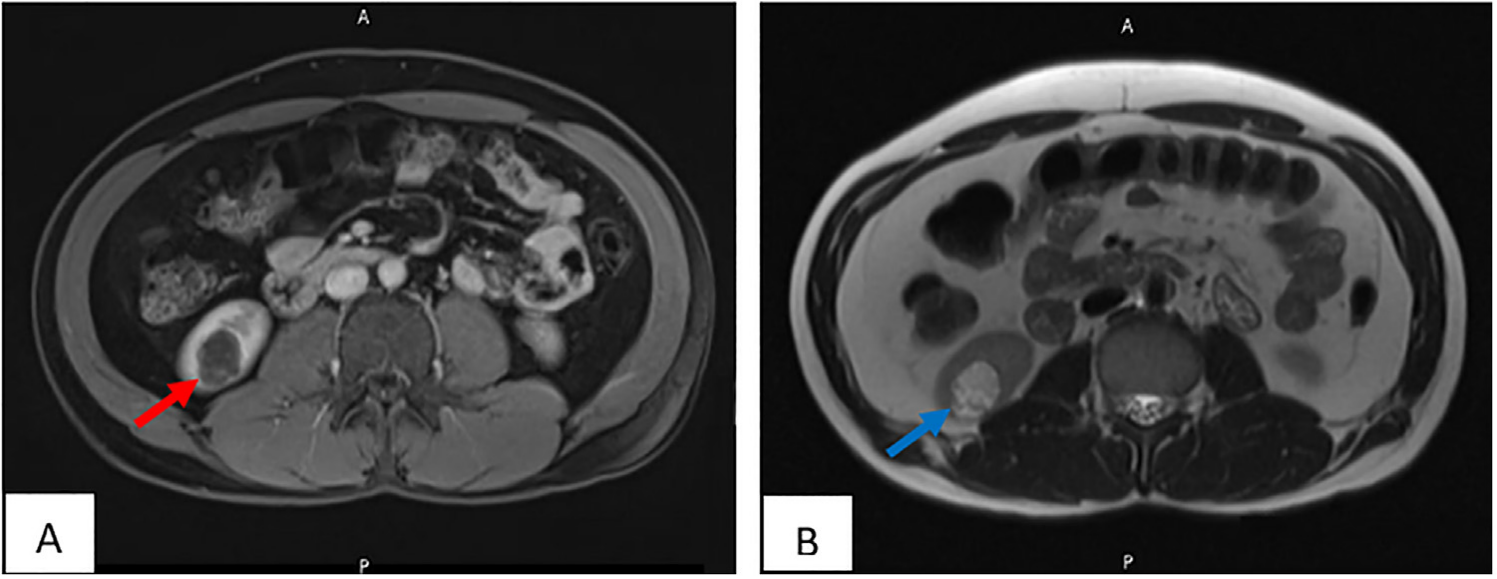
MRI of the right renal lesion, (A) low signal lesion on T1 sequence (red arrow), (B) hyperintense lesion on T2 (blue arrow).

A 35 year old gentleman previously healthy, presented with nonspecific abdominal pain, The pain was not associated with any other urinary symptoms. Physical examination, abdominal and genital examination were unremarkable. Laboratory tests were within normal ranges. Computed tomography (CT) scan of the abdomen and pelvis with IV contrast showed a well-defined right kidney lesion that is homogenous and hypodense without enhancement, measuring 22 × 27 × 18.6 mm with Hounsfield density of 17, there were no calcifications, septations or any other renal lesions bilaterally. Lesion was diagnosed as a right renal cortical cyst Bosniak class I (Fig. 1). A Follow up Abdominal ultrasound (US) 1 month after the CT scan showed a well-defined hyperechoic lesion seen in the left lower pole parenchyma measuring 26 × 18 × 27 mm (Fig. 2). The patient then underwent a magnetic resonance imaging (MRI) scan, showed parenchymal lesion in the lower pole of right kidney measuring 25 × 19.6 × 22 mm, showing low signal on T1 (Fig. 3A), high signal in T2 (Fig. 3B), showing internal nodular enhancement representing Bosniak class IV cyst. The patient underwent uneventful robotic assisted partial nephrectomy with warm ischemia time of 24 minutes.

**Fig. 4:**
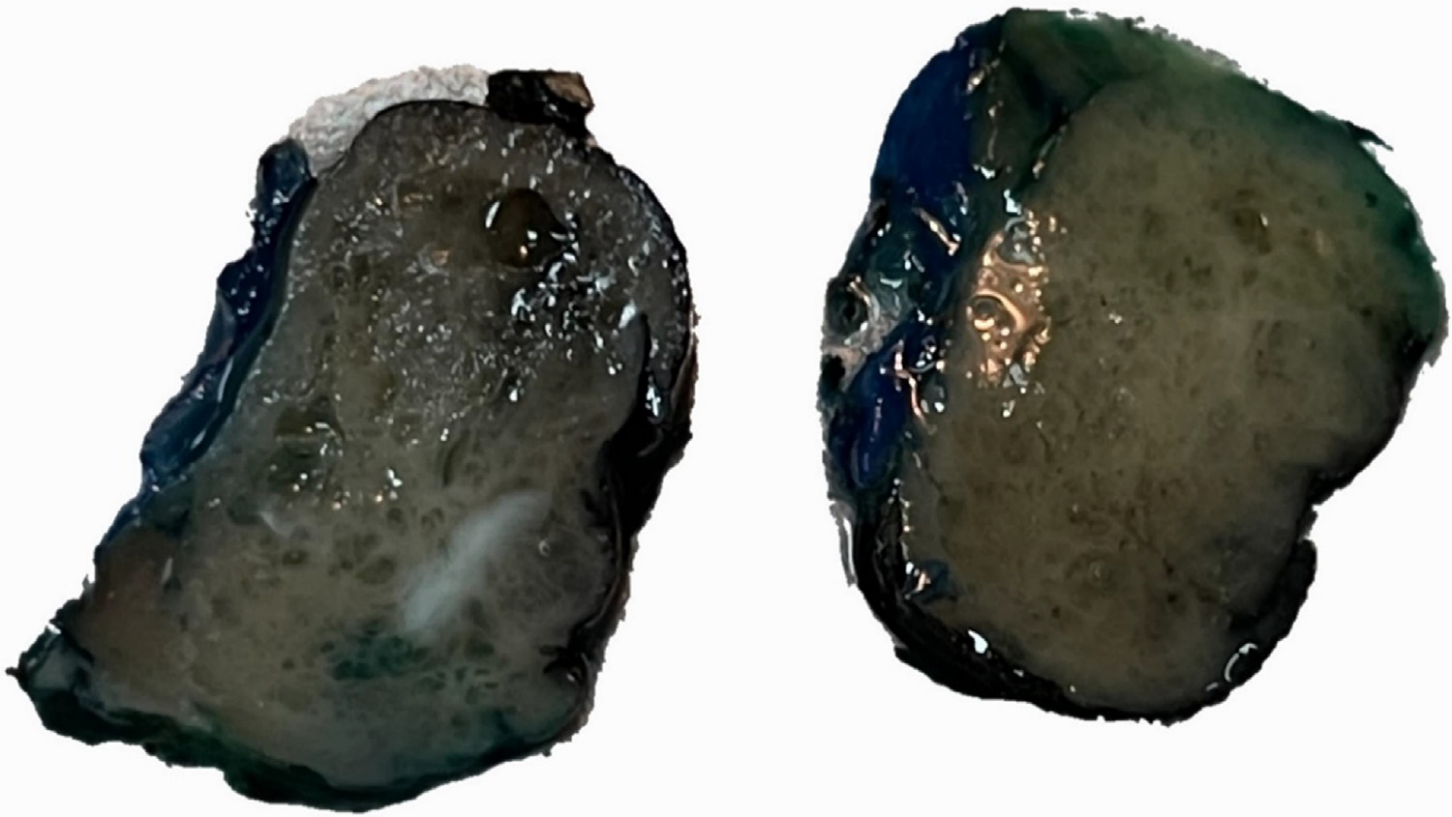
Gross photograph demonstrates a well-circumscribed tumor with a spongy cut surface and variable-sized cysts containing serous fluid.

**Fig. 5:**
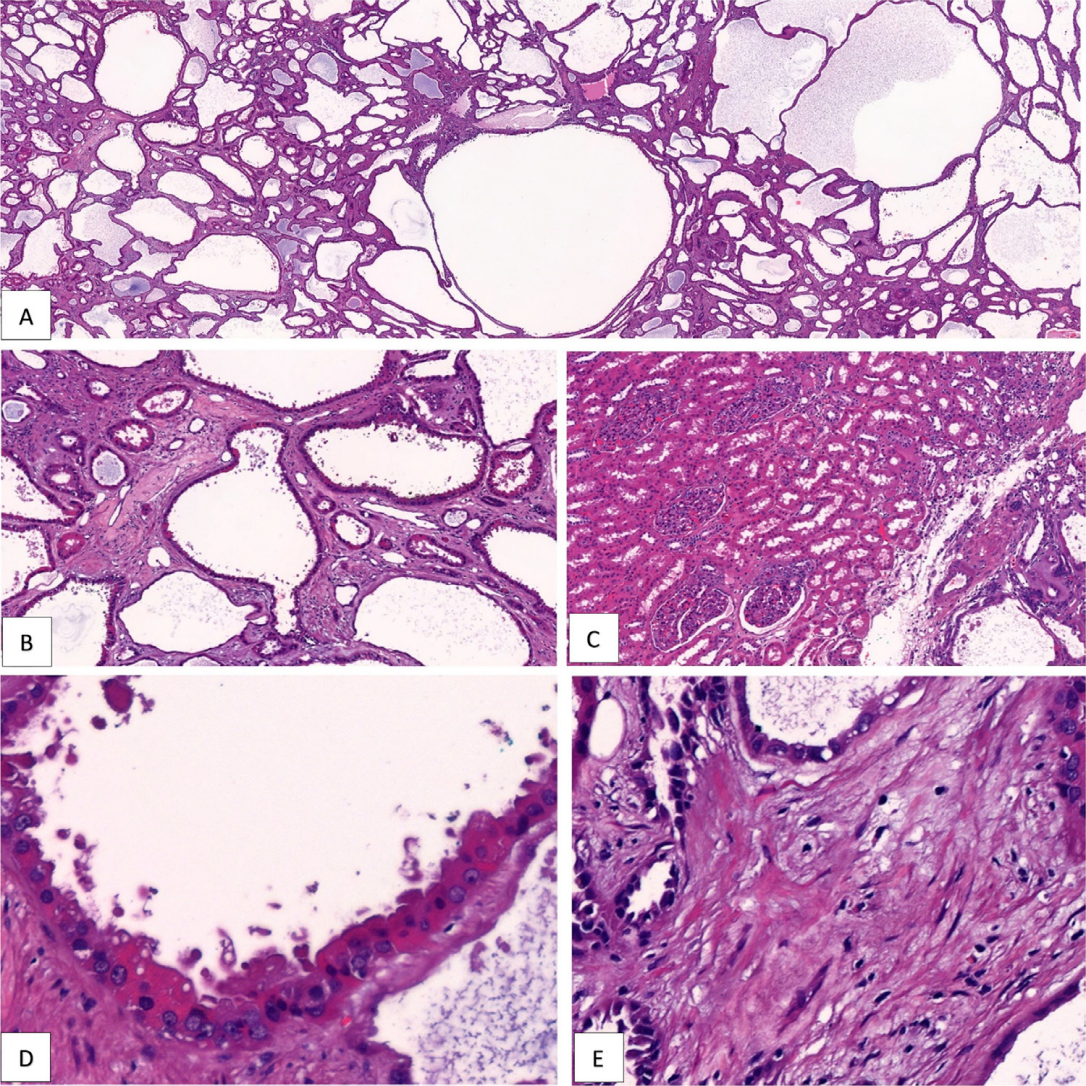
(A and B) Microscopy showed both cystic and tubular structures with intervening stroma (A: hematoxylin and eosin [H&E], magnification ×4; B: H&E, magnification ×10). (C) TCRCCs are well circumscribed with partial to incomplete encapsulation. In this case the tumor was in direct contact with normal appearing renal parenchyma,(C: H&E, magnification ×10). (D) TCRCC consists of tubules and variable-sized cysts lined by cells with abundant eosinophilic cytoplasm. Lining cells often have large nuclei with open chromatin and variably conspicuous nucleoli (D: H&E, magnification ×40). (E) Characteristically, the intervening stroma between the tubule and cysts in tubulocystic renal cell carcinoma is fibrotic and hypocellular (E: H&E, magnification ×40).

Histopathology; Gross examination demonstrated a 3 x 2 x 1 cm well-circumscribed tumor with a spongy cut surface and variable-sized cysts containing serous-watery fluid (Fig. 4). Microscopic examination demonstrated a well-circumscribed tumor characterized by small-to-intermediate sized, cystically dilated tubules. These tubules were lined by a single layer of variably attenuated to hobnailing cuboidal epithelial cells. The cells showed abundant eosinophilic cytoplasm and large nuclei, with irregular nuclear membranes, and conspicuous but not prominent nucleoli. Mitoses, tumor necrosis, and hemorrhage were not identified. The intervening stroma was fibrotic and hypocellular. No sarcomatoid or rhabdoid features were identified. No evidence of lymphovascular invasion or angioinvasion was seen. The tumor was predominantly un-encapsulated and was in direct contact with histologically normal renal cortical parenchyma (Fig. 5). The differential diagnoses for this case included tubulocystic carcinoma (TCRCC), mixed epithelial and stromal tumor (MEST), angiomyolipoma with epithelial cysts (AMLEC), acquired cystic disease-associated renal cell carcinoma (ACD-RCC), and oncocytoma with macrocystic architecture. MEST and AMLEC require the presence of cellular ovarian-type stroma and sometimes smooth muscle stroma, particularly in AMLEC. Our case lacked these features therefore, MEST and AMLEC were excluded. While ACD-RCC can show histologic overlap with TCRCC, ACD-RCC typically shows a more complex architecture and importantly, requires the presence of end-stage kidney disease with multiple cysts; which this patient did not have. Oncocytoma was excluded as it requires some areas of solid nested architecture and doesn't show diffuse tubulocystic architecture such as in this case.

## Discussion

Tubulocystic RCC that was in the WHO classification of 2016 constitutes approximately less than 1 percent of all renal cancers and individuals affected are in the age range of 30s to 90s, with a mean age of occurrence of 58 years [5]. Due to its relatively indolent clinical features, early recognition and treatment favorably with surgical options ensures a highly favorable prognosis and almost normal lifestyle postsurgery [2]. Although mostly asymptomatic, tubulocystic RCC may present with symptoms that include distention and pain of the abdomen and hematuria [6].

Since the tumor is incidentally found many of the times, it is crucial not to overlook the radiological features that this tumor can present with, providing a guide to radiologists and physicians to help formulate this diagnosis in mind once certain features are met on different radiological scans. On ultrasound, the tumor appears markedly hyperechoic with posterior acoustic enhancement [7]. The enhancement pattern is crucial as it helps in differentiating this tumor from other solid tumors of the kidney, such as angiomylipoma or RCC [7].

On CT scan, tubulocystic RCC appears as cystic lesions with Bosniak classifications ranges between grades I-IV and no or very little internal septations or calcifications, explaining the low Hounsefield units figures for this tumor [7]. With regards to MRI scans, it has been shown that it is more accurate than CT scans to evaluate the cystic structure and classification according to the Bosniak classification of cysts [8].

In terms of histopathological features of tubulocystic RCC, grossly, it has been described to be a well-defined multicystic lesion with a Swiss-cheese like appearance [9]. Microscopically, it consists of tubules and cysts that are well defined and surrounded by eosinophils with evident nucleoli [9]. In terms of immunohistochemical (IHC) staining, most tumor cells have been shown to stain positive for Vimentin, CD10, PAX8, and AMACR [10]. On the genetic level, it is thought that tubulocystic RCC has genetic features that are closely related to papillary RCC, however, with the help of other IHC, radiological and pathological features, it can help to distinguish tubulocystic RCC from other renal tumors [11]. We present a case of a male patient who was diagnosed with tubulocystic RCC with initial CT scan showing features Bosinak I renal cyst, and was diagnosed as Bosniak IV on MRI, the discordant radiological features add to the challenge in diagnosing such cases.

## Conclusion

In conclusion, tubulocystic RCC is a new variant of renal cancers that is known to have a dormant clinical course with no prominent symptoms. Although most cases are found incidentally on imaging, it usually has a low risk of metastasis and early detection is favored. Treatment usually is surgical and postsurgical course usually is unremarkable. Although tubulocystic RCC may thought to be related or similar to other renal tumors, certain radiological, pathological and genetic features aid in classifying this as a separate and solely existent entity.

## Ethical statement

This case has been reviewed and approved by the Institutional Review Board (IRB) under approval number MRC-04-24-370, ensuring adherence to ethical guidelines and participant safety protocols.

## Patient consent

A written consent for publication was taken from the patient according to our institution policy.

## References

[1] Alexiev BA, Drachenberg CB (2013). Tubulocystic carcinoma of the kidney: a histologic, immunohistochemical, and ultrastructural study. Virchows Arch.

[2] Xing S, Liu A, Yang X, Chen L, Xu D (2021). Tubulocystic renal cell carcinoma: Two-case report and literature review. Int J Immunopathol Pharmacol.

[3] Sarungbam J, Mehra R, Tomlins SA, Smith SC, Jayakumaran G, Al-Ahmadie H (2019). Tubulocystic renal cell carcinoma: a distinct clinicopathologic entity with a characteristic genomic profile. Mod Pathol.

[4] Banerjee I, Yadav SS, Tomar V, Yadav S, Talreja S (2016). Tubulocystic Renal Cell Carcinoma: A Great Imitator. Rev Urol.

[5] Khera S, Elhence P, Yadav T, Pandey H (2022). Tubulocystic Renal Cell Carcinoma: An Underrecognized Clinicopathologic Entity. Ochsner J.

[6] Bhullar JS, Varshney N, Bhullar AK, Mittal VK (2014). A New Type of Renal Cancer--Tubulocystic Carcinoma of the Kidney. A Review of the Literature. Int J Surg Pathol..

[7] Cornelis F, Hélénon O, Correas JM, Lemaitre L, André M, Meuwly JY, Sengel C (2016). Tubulocystic renal cell carcinoma: a new radiological entity. Eur Radiol.

[8] Honda Y, Nakamura Y, Goto K, Terada H, Sentani K, Yasui W (2018). Tubulocystic renal cell carcinoma: a review of literature focused on radiological findings for differential diagnosis. Abdom Radiol (NY).

[9] Zhou M, Yang XJ, Lopez JI, Shah RB, Hes O, Shen SS (2009). Renal tubulocystic carcinoma is closely related to papillary renal cell carcinoma: implications for pathologic classification. Am J Surg Pathol.

[10] Alexiev BA, Drachenberg CB (2013). Tubulocystic carcinoma of the kidney: a histologic, immunohistochemical, and ultrastructural study. Virchows Arch.

[11] Yang XJ, Zhou M, Hes O, Shen S, Li R, Lopez J (2008). Tubulocystic carcinoma of the kidney: clinicopathologic and molecular characterization. Am J Surg Pathol.

